# Is Vitamin B6 a Precision Therapy for Neonatal Seizures?

**DOI:** 10.3390/neurolint17100157

**Published:** 2025-10-01

**Authors:** Raffaele Falsaperla, Vincenzo Sortino, Bruna Scalia, Marco Andrea Nicola Saporito

**Affiliations:** 1 Department of Medical Science-Pediatrics, University of Ferrara, 44121 Ferrara, Italy; 2 Unit of Pediatrics and Pediatric Emergency, Azienda Ospedaliero-Universitaria Policlinico “Rodolico-San Marco”, 95121 Catania, Italy; sortino.vinci@gmail.com; 3 National Council of Research, Institute for Research and Biomedical Innovation (IRIB), Unit of Catania, 95126 Catania, Italy; 4 Neonatal Intensive Care Unit, Azienda Ospedaliero Universitaria Policlinico “G. Rodolico-San Marco”, 95121 Catania, Italy; b.scalia@hotmail.it (B.S.); marcosaporito@hotmail.com (M.A.N.S.)

**Keywords:** vitamin B6, neonatal seizures, pyridoxine-dependent epilepsy

## Abstract

**Background**: Neonatal seizures are critical neurological events with long-term implications for brain development. Standard antiseizure medications, such as phenobarbital, often yield suboptimal seizure control and may be associated with neurotoxicity. This narrative review explores the role of vitamin B6 as a precision therapy in neonatal seizure syndromes, particularly in pyridoxine-responsive conditions. **Methods**: We conducted a narrative review of the biochemical functions of vitamin B6, focusing on its active form, pyridoxal 5′-phosphate (PLP), and its role as a coenzyme in neurotransmitter synthesis. We examined the genetic and metabolic disorders linked to vitamin B6 deficiency, such as mutations in pyridox(am)ine 5’-phosphate oxidase (PNPO), Aldehyde Dehydrogenase 7 Family Member A1 (ALDH7A1), alkaline locus phosphatase (ALPL), and cystathionine β-synthase (CBS), and discussed the clinical rationale for empirical administration in acute neonatal seizure settings. **Results**: Vitamin B6 is essential for the synthesis of gamma-aminobutyric acid (GABA), dopamine, and serotonin, with PLP-dependent enzymes such as glutamic acid decarboxylase and aromatic L-amino acid decarboxylase playing central roles. Deficiencies in PLP due to genetic mutations or metabolic disruptions can result in treatment-resistant neonatal seizures. Early supplementation, especially in suspected vitamin B6-dependent epilepsies, may provide both diagnostic clarity and seizure control, potentially reducing exposure to conventional antiseizure medications. **Conclusions**: Vitamin B6-responsive epilepsies highlight the clinical value of mechanism-based, individualized treatment approaches in neonatology. Incorporating genetic and metabolic screening into seizure management may improve outcomes and aligns with the principles of precision medicine.

## 1. Introduction

Neonatal seizures represent one of the most challenging emergencies in neonatal medicine, with an incidence ranging from 1 to 3 per 1000 live births [1]. These seizures are not merely transient episodes of aberrant electrical activity but may signify critical events with long-lasting implications for the infant’s neurodevelopmental trajectory [2]. The etiologies are diverse, encompassing hypoxic–ischemic encephalopathy, inborn errors of metabolism, and monogenic epileptic syndromes [2]. Prompt and effective management is essential to mitigate the risk of secondary brain injury and long-term neurodevelopmental impairment [3].

Despite decades of use, conventional antiseizure medications (ASMs) such as phenobarbital remain suboptimal. Phenobarbital achieves seizure control in only approximately 40–50% of neonates and is associated with adverse neurodevelopmental and sedative effects [3,4]. Reflecting these limitations, the 2024 report by the International League Against Epilepsy (ILAE) on neonatal seizure management emphasized the need for alternative, mechanism-targeted therapies, particularly for treatable metabolic epilepsies such as pyridoxine-responsive seizures [4].

Recent advances in neonatal intensive care and the emergence of precision medicine have revitalized interest in vitamin B6 (pyridoxine) as a potential first-line therapy for specific neonatal seizure syndromes [5]. This is particularly relevant given the expanding recognition of pyridoxine-dependent epilepsy (PDE) and related disorders, which are now identifiable via rapid genetic and metabolic screening. The early identification of such cases allows for targeted therapy, potentially reducing cumulative ASM exposure and minimizing associated iatrogenic risks.

Over the past two decades, the field of vitamin B6-dependent epilepsies has evolved and several advances in the understanding of vitamin B6’s role, functioning, metabolism, genetics and related conditions have been provided. The current literature is still scattered across case reports, small case series and limited observational studies; furthermore, many neonates with vitamin B6-responsive seizures are misdiagnosed or treated with conventional ASMs and this, unavoidably, delays effective treatment [3,5].

Vitamin B6 is a simple, well-tolerated, and essential cofactor in neurotransmitter biosynthesis, notably in the GABAergic pathway, and thus it emerges as a promising candidate within a precision medicine framework. While its administration in PDE can be life-saving, early diagnosis remains a challenge due to the lack of pathognomonic clinical features [6]. In this context, empirical pyridoxine trials may serve as a pragmatic diagnostic and therapeutic tool, facilitating timely seizure control and improving long-term neurodevelopmental outcomes.

We believe that our narrative review offers a valuable update on the current literature and presents a comprehensive and novel perspective on the latest evidence regarding the role of vitamin B6 in neonatal seizures. This includes a detailed focus on recent advances—such as its biochemical mechanisms, clinical uses, and implications for personalized treatment approaches—as well as future directions involving early diagnosis and individualized therapies. Our work seeks to integrate the previously fragmented studies, and underscore key clinical signs, EEG patterns, biochemical markers, and genetic testing methods to enhance early detection and management. Additionally, it emphasizes the need for increased awareness and the establishment of guidelines, supports the creation of diagnostic algorithms, and thus serves as an important resource for neonatologists, pediatric neurologists, geneticists, and other healthcare professionals involved in neonatal care.

## 2. The Biochemical Rationale: Why Vitamin B6 Is a Big Deal

To comprehensively investigate the role of Vitamin B6 in neonatal seizures, we performed a narrative review of the literature sourced from medical electronic databases such as PubMed Central, Cochrane Library, Medline, Scopus, and Web of Science. The following search terms were used: “vitamin b6” and “newborns” or “neonatal seizures”, “vitamin b6” and “epilepsy”, “vitamin b6” and “deficiency”, “pyridoxine-dependent epilepsy” and “neonatal seizures”. All the selected studies described neonatal patients with seizures responsive to vitamin B6; filters applied were English language and human studies, and both empirical research and review articles were included to contextualize our findings. We screened titles and abstracts of 276 articles published between January 2015 and January 2025 and excluded those with exclusionary medical content according to our selection criteria. Finally, we analyzed the subset of 56 articles and selected 27 articles to include in the present review, including 3 case reports, 9 cohort studies and 15 review articles.

Findings from the literature highlight how vitamin B6, in its biologically active form pyridoxal 5’-phosphate (PLP), is a vital cofactor for over 140 enzymatic reactions in the human body, with a significant proportion of these involved in amino acid metabolism and neurotransmitter synthesis. Its role in the central nervous system (CNS) is particularly crucial, as PLP-dependent enzymes regulate the synthesis of key neurotransmitters that maintain the delicate balance between neuronal excitation and inhibition—an equilibrium essential for normal brain function and particularly vulnerable during the neonatal period [6].

Two major PLP-dependent enzymes critical in neurotransmission include:

1. Glutamic Acid Decarboxylase (GAD): GAD catalyzes the decarboxylation of glutamate, the principal excitatory neurotransmitter in the brain, into GABA, the main inhibitory neurotransmitter. The GABAergic system plays a fundamental role in dampening neuronal excitability and preventing hyperexcitability that can manifest as seizures. In neonates, GABA’s function is developmentally regulated; while it initially has excitatory effects, it gradually becomes inhibitory as the brain matures, making proper PLP-dependent GAD function crucial in early life to prevent seizure susceptibility.
2. Aromatic L-Amino Acid Decarboxylase (AADC): This enzyme catalyzes the decarboxylation of 5-hydroxytryptophan to serotonin and L-DOPA to dopamine—neurotransmitters involved in regulating mood, arousal, motor function, and neurodevelopment. Impaired AADC activity due to vitamin B6 deficiency can disrupt these pathways, potentially contributing to neurodevelopmental deficits beyond seizure activity.

Vitamin B6 deficiency, whether due to genetic defects in vitamin B6 metabolism or dietary insufficiency, leads to reduced PLP availability. This impairs GAD and AADC activities, resulting in decreased synthesis of GABA and monoamines. The net effect is a shift towards excitatory neurotransmission, which lowers the seizure threshold and predisposes neonates to recurrent seizures [6].

Moreover, PLP is involved in other enzymatic reactions relevant to neuroprotection and metabolism, including:

1. Serine hydroxymethyltransferase (SHMT): Involved in one-carbon metabolism, affecting DNA synthesis and methylation, processes important in brain development.
2. Cystathionine β-synthase (CBS): Catalyzes the conversion of homocysteine to cystathionine, with implications for homocysteine neurotoxicity and oxidative stress.

Given these multifaceted roles, vitamin B6 deficiency can produce a wide range of neurological symptoms, with seizures often being the earliest and most prominent manifestation in neonates. This biochemical rationale provides the foundation for using vitamin B6 supplementation as a precision therapy in neonatal seizures, particularly in PDE and related disorders where mutations impair vitamin B6 metabolism or function [5,6]. Recent preclinical evidence has suggested a higher susceptibility of female rats to the neuroprotective effect induced by vitamin B6 for seizure cessation, but found no confirmation in clinical experiences, so far; in the optics of a super-precision therapy, future perspectives might focus on such aspects as well [7].

## 3. Vitamin B6 Metabolic Pathway

Vitamin B6 exists in several vitamers, including pyridoxine, pyridoxal, and pyridoxamine, which can be bioactivated through enzymatic processes into the biologically active form, PLP. This conversion is essential for the coenzyme function of PLP in neurotransmitter synthesis and numerous neuronal metabolic pathways [6,8].

The metabolic activation pathway involves the following enzymatic steps:

1. Pyridoxine PN is first oxidized to pyridoxal by pyridoxine 5’-oxidase.
2. Pyridoxal PL is then phosphorylated by pyridoxal kinase to form pyridoxal 5’-phosphate (PLP), the active coenzyme form.

This conversion is tightly regulated and crucial for maintaining adequate PLP levels in neurons, where it facilitates enzymatic reactions such as decarboxylation and transamination necessary for neurotransmitter biosynthesis [8].

Deficiencies or genetic mutations affecting enzymes in this pathway, such as PNPO deficiency or mutations in ALDH7A1, disrupt PLP synthesis or availability, leading to neurological manifestations including neonatal seizures [6,9]. Understanding this metabolic pathway is fundamental for diagnosing and targeting treatments in vitamin B6-dependent epilepsies.

**Figure 1:**
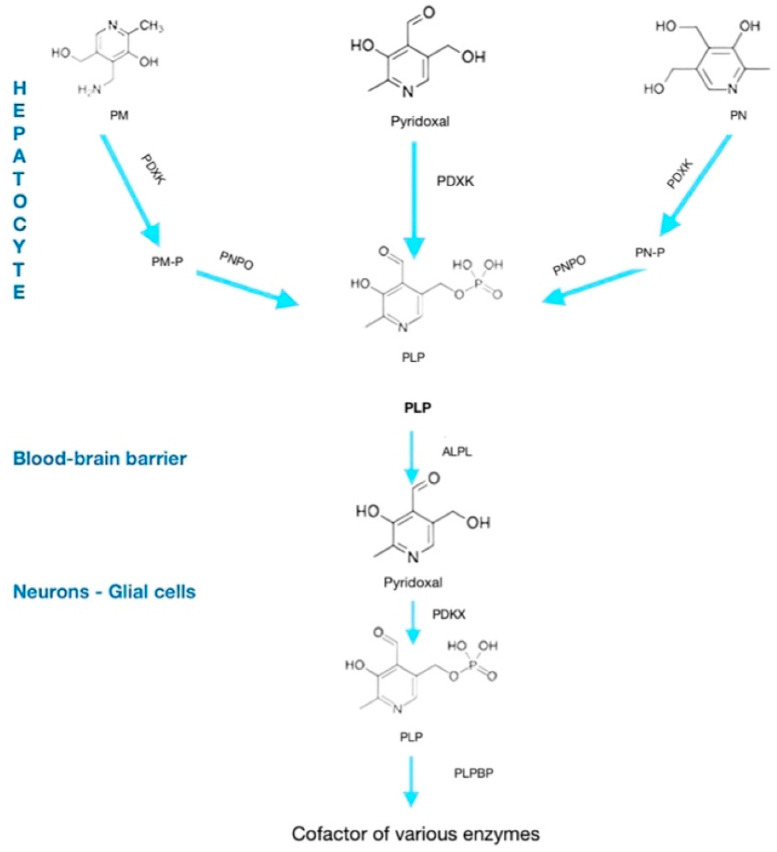
Metabolic pathway of vitamin B6. PM, pyridoxamine; PN, pyridoxine; PDXK, pyridoxal-kinase; PNPO, pyridoxamine 5-P oxidase; PLP, pyridoxal-phospate; ALPL, alkaline phosphatase, tissue-nonspecific isoenzyme; PLPBP, PLP-binding protein.

The metabolic pathway of Vitamin B6 is reported in Figure 1.

PLP is the active coenzyme form of vitamin B6 and is indispensable in the synthesis, metabolism, and regulation of multiple neurotransmitters critical for central nervous system function. Acting as a versatile coenzyme, PLP facilitates a wide range of enzymatic reactions including decarboxylation, transamination, racemization, and β-elimination that are pivotal in neurotransmitter biosynthetic pathways [8,9].

In particular, PLP serves as a cofactor for:

1. GAD: PLP enables the conversion of glutamate GABA, the primary inhibitory neurotransmitter in the brain that reduces neuronal excitability and prevents hyperexcitability linked to seizures [6].
2. AADC: PLP assists in converting L-DOPA to dopamine and 5-hydroxytryptophan to serotonin, neurotransmitters essential for mood regulation, motor control, and cognitive function.
3. SHMT: Involved in one-carbon metabolism, PLP-dependent SHMT plays a role in producing neurotransmitter precursors and maintaining cellular methylation status, which affects gene expression and neurodevelopment.
4. CBS: PLP-dependent CBS regulates homocysteine metabolism, preventing the accumulation of neurotoxic metabolites that can influence seizure susceptibility.

Because PLP participates directly in these enzymatic activities, its deficiency disrupts neurotransmitter balance, leading to excitatory/inhibitory imbalance in the brain. This imbalance is a key pathogenic mechanism in many neonatal seizure disorders, including PDE and PNPO deficiency. Furthermore, PLP is involved in modulating neurotransmitter receptor function and gene expression, emphasizing its broad neurochemical importance beyond classical enzymatic roles [8,9]. The critical dependence of neonatal brain function on PLP underscores the rationale for timely vitamin B6 administration in suspected cases of neonatal seizures with metabolic etiologies [6,8,9].

**Figure 2:**
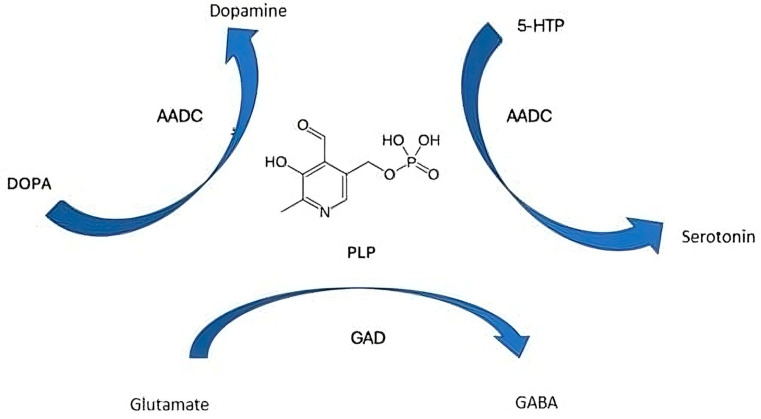
The neurotransmitter synthesis with pyridoxal 5′-phosphate as a coenzyme. 5-HTP, 5-hydroxytryptophan; PLP, pyridoxal phosphate; GAD, glutamic acid decarboxylase; AADC, aromatic L-amino acid decarboxylase.

The neurotransmitter synthesis with PLP as a coenzyme is reported in Figure 2.

PLP’s role in numerous metabolic pathways helps explain why various molecules from these pathways have been explored as potential biomarkers for certain disorders. Currently, biochemical tests measuring metabolites such as α-aminoadipic semialdehyde (α-AASA), Δ’-piperidine-6-carboxylic acid (P6C), and pipecolic acid (PA) in urine, plasma, and cerebrospinal fluid (CSF) have been conducted in patients exhibiting clear symptoms of PDE [10,11]. However, it should be noted that these biomarkers—linked to ALDH7A1-related PDE—are not entirely specific, as they may be elevated in other conditions. Additionally, their analysis can be challenging and inconsistent, and the diagnostic tools for their measurement are not widely available.

More recently, new molecules such as 6-oxopiperidine-2-carboxylic acid (6-oxo-PIP) and the 2S,6S- and 2S,6R-oxopropylpiperidine-2 compounds (2-OPP) have been identified as promising biomarkers [12]. The former is considered a stable marker accumulating in blood, urine, plasma, and CSF; it remains stable at room temperature and is compatible with newborn screening methods using liquid chromatography–tandem mass spectrometry (LC-MS/MS). The latter was discovered through untargeted metabolomics in combination with infrared ion spectrometry and shows promise as a biomarker detectable in dried blood spots, with potential for large-scale newborn screening applications.

## 4. Vitamin B6 Metabolic and Clinical Features

Vitamin B6 represents a paradigmatic example of precision therapy in neonatal seizures, where targeted supplementation can significantly improve seizure control and long-term neurological outcomes [13]. This approach is particularly valuable given the heterogeneous and often nonspecific clinical presentation of PDE and related vitamin B6 metabolism disorders, which complicates early diagnosis. Due to the absence of pathognomonic clinical features and the potential severity of untreated seizures, the empirical administration of vitamin B6 is recommended in neonatal acute seizure settings. This strategy not only serves as a therapeutic intervention but also facilitates differential diagnosis by identifying neonates who respond to pyridoxine, thereby optimizing subsequent clinical management [13,14,15]. PDE is caused by genetic mutations affecting enzymes involved in vitamin B6 metabolism, such as ALDH7A1, which encodes antiquitin, and PNPO, encoding pyridox(am)ine 5’-phosphate oxidase. These defects lead to functional vitamin B6 deficiency in the CNS, despite normal systemic levels, resulting in refractory seizures [9]. ALDH7A1-PDE is the most characterized epileptic disorder caused by pyridoxine deficiency, with more than 100 mutations identified to date. Deficiency of the ALDH7A1 enzyme disrupts lysine metabolism, leading to the accumulation of upstream compounds that result in PLP depletion/inactivation. Seizures typically start in the neonatal-perinatal period or early infancy, often within hours or days of birth, and may include tonic–clonic, myoclonic or focal seizures. Atypical or late-onset forms are reported. Seizures are often, but not always, refractory to common ASMs and show a dramatic response to pyridoxine. Even when seizures are controlled rapidly with pyridoxine, though, neurodevelopmental outcomes are often unpredictable and many patients suffer developmental delay and intellectual disability. Diagnosis relies on clinical suspicion, biochemical markers (α-AASA in urine, plasma or CSF), elevated P6C, pipecolic acid and newer biomarkers (see above). Indeed, genetic testing is mandatory to confirm diagnosis: WES or targeted panels are used. Treatment relies on pyridoxine administration, lysine restricted diet and arginine supplementation [16,17,18,19,20].

PNPO deficiency is caused by mutations in the PNPO gene that encodes for pyridox(am)ine-5’-phosphate oxidase, vital for converting dietary B6 vitamers to active PLP. Clinical presentation includes neonatal-onset epileptic encephalopathy with seizures starting within hours to days after birth; these seizures often do not respond to standard ASMs but typically improve with PLP and, occasionally, with pyridoxine treatment. Additional neonatal presentation may also include fetal distress, hypoglycemia, acidosis and a distinctive burst-suppression pattern on the EEG. Since there are no consistent biochemical markers, diagnosis relies on clinical suspicion that must be confirmed by genetic testing, showing pathogenic variants in the PNPO gene and by therapeutic trials, initially with PLP and subsequently with pyridoxine. This often results in a dramatic seizure cessation within hours. The condition exhibits genotype–phenotype correlations with severe mutations (such as nonsense, frameshift mutations) leading to early-onset PLP-only responsive conditions, and milder mutations (such as missense) leading to milder phenotypes and seizures partially responding to other ASMs as well. Early diagnosis and prompt PLP administration are essential for effective seizure management and improved long-term outcomes [16,20]. 

In addition to PDE, other neurological conditions are linked to vitamin B6 deficiency and include nutritional deficiency states, certain inborn errors of metabolism, and drug-induced deficiency (such as isoniazid toxicity) [21,22]. Among inherited metabolic disorders associated with vitamin B6 metabolism, hypophosphatasia caused by mutations in the ALPL gene disrupts tissue-nonspecific alkaline phosphatase activity, which is required for the dephosphorylation of pyridoxal 5’-phosphate to pyridoxal, the form that crosses the blood–brain barrier. This impairment leads to decreased CNS vitamin B6 availability and can manifest with seizures and neurological symptoms in affected neonates [18,19], along with bone abnormalities (short limbs, poor ossification). Biochemical features include elevated serum PLP and markedly decreased serum alkaline phosphatase. Seizures often respond to intra-venous or oral pyridoxine (50–100 mg/day, adjusted based on clinical response). Despite high serum PLP levels, neuronal B6 is low, so supplementation is effective. Bone deformities and long-term neurological outcomes may improve with enzyme replacement therapy (ERT) with asfotase alfa. Seizures are typically responsive to pyridoxine; bone deformities may improve with enzyme-replacement therapy (asfotase alfa) [16,20,23,24].

Similarly, homocystinuria, due to mutations in the CBS gene encoding cystathionine β-synthase, results in impaired homocysteine metabolism and secondary vitamin B6 deficiency. This deficiency exacerbates neurological dysfunction and seizure susceptibility through the disruption of neurotransmitter synthesis and increased neurotoxicity from homocysteine accumulation, which results in toxicity to blood vessels, nerves and connective tissue. CBS can be classified into responsive to pyridoxine (approximately 50% of cases), caused by milder mutations affecting cofactor binding or folding, often associated with milder phenotypes and later onset and responding to high-dose pyridoxine (100–500 mg/day), and pyridoxine-unresponsive CBS deficiency caused by severe enzyme defects, requiring dietary methionine restriction. Clinical presentation varies widely and, if untreated, can include developmental delay, intellectual disability, marfanoid habitus, ectopia lentis, thromboembolic events and osteoporosis. Responsive patients can have minimal to no symptoms if treated, hence emphasizing the need for early diagnosis and treatment [16,20].

Table 1 shows features of the diseases associated with vitamin B6 metabolism-disorders. 

Given the critical role of vitamin B6 in neurotransmitter synthesis and neuronal function, timely identification and treatment are essential to prevent irreversible neurological damage and improve neurodevelopmental trajectories in affected infants.

Current treatment approaches, as detailed in *Treatment of Seizures in the Neonate: Guidelines and Consensus-Based Recommendations—Special Report from the ILAE Task Force on Neonatal Seizures*, support a stepwise methodology [20]. These guidelines recommend phenobarbital as the initial therapy of choice for seizure control, followed by the use of alternative antiseizure medications (ASMs) in cases where seizures prove refractory [14,25]. Nevertheless, it is important to emphasize that although phenobarbital may effectively suppress seizures, it does not rule out underlying etiologies that may necessitate targeted precision treatments. Among these alternatives, precision therapies include pyridoxine (vitamin B6) for pyridoxine-dependent epilepsy and specific interventions for metabolic disorders identified through biochemical screening [26]. Precision therapies represent a paradigm shift in medical treatment because they are designed to specifically address the underlying molecular or genetic causes of a disease. By tailoring interventions to the individual patient’s unique biological profile, these therapies have the potential to not only halt or slow disease progression but also to fundamentally modify its natural course. This targeted approach enhances therapeutic efficacy, minimizes adverse effects, and ultimately leads to significantly improved clinical outcomes, embodying the core principles of precision medicine [27]. Pyridoxine, in particular, serves as an early and effective example of precision therapy, demonstrating the significant impact such treatments can have on the disease’s natural history [28]. Without timely and precise therapeutic intervention, PDE may result in significant mortality and severe long-term complications [14]. Therefore, early and accurate diagnosis is essential not only to reduce the risk of fatal outcomes but also to enhance neurodevelopmental trajectories, including psychomotor skills and language acquisition, thereby improving the overall quality of life for affected patients [14].

PDE is classified into two primary types, classical and atypical, each with distinct clinical presentations and diagnostic challenges. The classical form of PDE generally emerges within the first weeks or months after birth, most commonly presenting as neonatal seizures that do not respond to standard antiepileptic treatments [29]. These seizures can manifest as a variety of types, including partial or generalized seizures, clonic, myoclonic, tonic seizures, and infantile spasms [30]. Additionally, some affected infants experience seizures triggered by febrile illnesses. Notably, some mothers report abnormal fetal movements starting from the second trimester of pregnancy, suggesting that the neurological dysfunction in PDE may begin prenatally [31]. Without prompt and appropriate treatment, classical PDE often progresses to recurrent status epilepticus and prolonged seizure episodes, frequently accompanied by abnormal facial expressions and unusual eye movements [31]. Generalized motor seizures were the most frequently reported initial seizure type, followed by focal motor seizures. Other presentations are complex presentations, including both focal and generalized manifestations, clonic, tonic–clonic, myoclonic seizures and epileptic spasms. Of interest, no particular seizure type demonstrated a marked predominance throughout the follow-up period [32]. Electroencephalographic (EEG) recordings in affected infants may show typical seizure activity even when overt seizures are not clinically apparent, indicating ongoing subclinical epileptiform discharges. The most frequently reported specific EEG pattern was burst-suppression/suppression-burst, mainly observed in patients with PLPB, ALDH7A1, and PNPO deficiencies. Hypsarrhythmia was also noted, particularly in those with PNPO deficiency. Paroxysmal interictal abnormalities were common, while a notable portion of patients exhibited non-paroxysmal EEG abnormalities, such as disorganized background activity and slowing. Some patients showed no interictal EEG abnormalities. Following the initiation of vitamin B6 treatment, many patients demonstrated no significant EEG abnormalities. Nevertheless, burst suppression and hypsarrhythmia persisted in a small subset of cases, and paroxysmal abnormalities remained present in several patients [32].

Infantile spasms accompanied by hypsarrhythmia represented a less frequent initial electroclinical presentation across all vitamin B6-related epilepsies. This finding supports the idea that pyridoxine or PLP supplementation may be more appropriate as a second-line option in such cases, following treatment with adrenocorticotropic hormone (ACTH) and vigabatrin [33,34]. Recently, epileptic spasms without associated hypsarrhythmia have been described in a 5-month-old infant who exhibited combined paroxysmal eye movement abnormalities and carried a novel bi-allelic pathogenic variant in the PLPBP gene [35].

Atypical clinical presentations resembling Dravet syndrome were noted in two individuals, although additional information regarding their clinical progression and therapeutic response was not reported [16]. A notable number of patients, particularly those with ALDH7A1 deficiency, experienced febrile seizures or repeated episodes of status epilepticus during febrile illnesses. These occurrences were generally not mitigated by temporary increases in pyridoxine dosage during maintenance therapy [16,17].

Although uncommon in B6-dependent epilepsy, absence seizures and atonic seizures were observed in a small subset of patients. Status epilepticus accounted for approximately 8% of initial presentations among individuals with a confirmed genetic diagnosis. While focal clonic status epilepticus was previously considered rare, its frequency appears comparable to other types.

Cognitive impairment is a common and significant concern in PDE, with intellectual disabilities particularly affecting expressive language abilities [17,35]. The severity of these neurological impairments is closely linked to the age at which symptoms first appear—earlier onset is generally associated with poorer neurodevelopmental outcomes. Furthermore, delays in diagnosis and initiation of treatment can exacerbate cognitive deficits. Remarkably, infants who receive antenatal pyridoxine therapy exhibit notably better intellectual outcomes and higher IQ scores compared to their untreated siblings, highlighting the crucial role of early intervention in modifying disease trajectory. However, there are documented cases of patients maintaining normal intellectual function despite the diagnosis, indicating some variability in disease expression and progression.

Atypical PDE, on the other hand, presents a more heterogeneous and diagnostically complex clinical picture. This form usually begins after the neonatal period, often manifesting after two months of age, although in rare cases onset can be delayed until adolescence, with diagnosis sometimes only achieved in early adulthood [18]. Unlike the classical form, seizures in atypical PDE may initially be unresponsive to pyridoxine but can become controlled with continued therapy over several months. Patients may also demonstrate transient improvement with conventional antiepileptic drugs before these become ineffective. Interestingly, seizure-free intervals lasting several months can occur following the cessation of pyridoxine treatment. A notable feature distinguishing atypical PDE is the responsiveness to folinic acid in some infants who fail to improve with pyridoxine alone in the early stages. Similar to classical PDE, the timing of symptom onset remains an important prognostic factor, with later onset generally correlating with better cognitive and developmental outcomes.

The effective treatment of PDE in children requires lifelong supplementation with pyridoxine, which is the first-line therapy. While it controls seizures, developmental delay and intellectual disability persist in at least 75% of cases, prompting the addition of complementary therapies [19].

Due to the rarity of the condition, controlled pediatric dosing trials are lacking, but current guidelines recommend 100 mg/day for newborns and 30 mg/kg/day (max 300 mg/day) for infants [6]. For infants presenting with acute seizures, an intravenous bolus of 100 mg of pyridoxine is advised, though this carries a risk of apnea and must be administered under monitoring with access to respiratory support and, if possible, EEG [20].

Breakthrough seizures can occur during febrile illnesses in children. In such cases, doubling the dose for the first 72 h is recommended [6].

Although generally safe, high-dose pyridoxine may cause reversible peripheral neuropathy, making regular clinical screening and electrodiagnostic testing necessary, especially for those receiving >500 mg/day [6].

Prenatal supplementation with 100 mg/day of pyridoxine is recommended for pregnancies at risk of PDE. This approach has been shown to prevent seizures and, in some genotypes, support normal development. Supplementation should begin early in gestation and be discontinued if genetic testing at 11–12 weeks excludes antiquitin (AQT) mutations. No adverse fetal effects have been reported [36].

In children unresponsive to pyridoxine, PLP should be considered. Non-responsiveness is typically due to PNPO deficiency, though idiopathic cases exist. The suggested dose is 30 mg/kg/day, noting that high PLP levels may cause liver dysfunction and seizures [6,20,36].

If seizures persist or are partially responsive, folic acid may be added, despite the mechanism being unclear. The recommended dose is 3–5 mg/kg/day, though high doses may worsen seizures [6,20].

In AQT deficiency, which causes an accumulation of toxic lysine metabolites, pyridoxine alone controls seizures, but metabolite levels remain high [20]. A lysine-restricted diet can help lower these levels and may improve neurodevelopmental outcomes. Thus, it is considered an adjunctive therapy [6,20].

For dietary control, lysine-free amino acid formulas are used. However, since they are also low in tryptophan, supplementation may be needed to avoid deficiency symptoms [19,20,37]. If formulas are unavailable or poorly tolerated, a natural protein-restricted diet is an alternative [19].

In addition, arginine supplementation has been shown to reduce lysine transport into the brain, potentially lowering toxic metabolites in cerebrospinal fluid (CSF). Arginine at 200 mg/day has been associated with improvements in motor and language function in pediatric patients, both alone and in combination with dietary therapy [6,20].

Table 2 shows the clinical features of vitamin B6-related neonatal epilepsies.

Table 3 shows the treatment strategies for vitamin B6-responsive neonatal seizures.

## 5. Discussion

Workup and proper treatment for neonatal seizures still represent some of the most challenging emergencies facing clinicians in neonatal medicine [1,3,4].

Recent advances in neonatal intensive care and the paradigm shift towards precision medicine have renewed focus on vitamin B6 as a potential first-line treatment for specific neonatal seizures syndromes. Vitamin B6-dependent epilepsies thus constitute a crucial, albeit often underestimated, niche of etiologies underlying neonatal seizures [4,5].

This narrative review aims to provide an update and to elucidate the multifaceted roles of vitamin B6, highlighting its participation in diverse metabolic pathways and enzymatic reactions, as well as its clinical implications for individualized therapeutic approaches and future research directions.

Vitamin B6 serves as a cofactor for numerous enzymatic processes, notably those involved in neurotransmitter synthesis, including GABA, serotonin and dopamine. Disruption in any of these vitamin B6-dependent pathways results in the accumulation of neurotoxic metabolites, precipitating refractory seizures typically with neonatal or perinatal onset [8,9].

Beyond classic ALDH7A1 mutations causing (PDE) and pyridox(am)ine-5’-phosphate oxidase (PNPO deficiency), the recent characterization of additional metabolic disorders—including hypophosphatasia and homocystinuria—has broadened the spectrum of vitamin B6-dependent epileptic syndromes [6].

The variability in clinical presentation reflects the variability in the underlying genetic etiologies and poses significant diagnostic challenges.

The prompt identification of vitamin B6-dependent epilepsies is vital given the dramatic response to early treatment in most cases. Multiple experiences, in fact, have reported a dramatic seizure cessation within minutes to hours following intravenous pyridoxine administration in affected neonates; pyridoxine administration is not devoid of risks, including apnea, so its use should be limited to intensive care setting. Along with “typical” cases, some “atypical” ones have been reported with unpredictable responses to vitamin B6 [5,9,10,13].

Clinically, vitamin B6-dependent epilepsies lack pathognomonic signs, frequently mimicking other neonatal encephalopathies such as HIE, thus impeding early recognition [9].

The advent of rapid whole-genome sequencing (WES) has, indeed, revolutionized diagnostic algorithms, complemented by recently identified high-sensitivity and high-specificity biomarkers detectable in serum, urine and CSF [10].

However, it should be acknowledged that access to the aforementioned tools (such as genetic testing, molecular diagnostics) remains limited in most clinical settings, thereby inevitably contributing to heterogeneity in clinical approaches.

Despite effective seizure control with vitamin B6 supplementation, long-term neurodevelopmental outcomes exhibit considerable variability, driven predominantly by underlying genetic factors. Ongoing research into genotype–phenotype correlations remains essential to elucidate prognosis and optimize management strategies [29,30]. Also, questions on the extent of prenatal, irreversible neuronal injury prior to treatment initiation and on the presence of unidentified underlying modifying genetic factors should be raised.

Dosing strategies widely differ among studies according to single-center experiences and the availability of intensive care [14,15].

The present work enlightens the complex molecular mechanisms and clinical features beyond vitamin B6-dependent epilepsies, also highlighting the need for some issues to be further clarified. The main current limitations emerging from the present work on the approach to vitamin B6-dependet epilepsies include heterogeneity in diagnostic tools, protocols and dosing regimens among centers. Future investigation should focus on better defining atypical and late-onset B6-responsive phenotypes, defining genotype–phenotype correlation, integrating rapid genetic diagnostics, advance biomarker discovery and feasibility in clinical practice, optimizing and standardizing dosing regimens, providing evidence-based internationally recognized protocols and establishing international registries to collect phenotypic, genetic and outcome data.

## 6. Conclusions

Neonatal seizures are often a race against time. Each episode carries the risk of inflicting lasting injury on the developing brain, making early and effective intervention imperative. In this context, vitamin B6 is emerging not merely as a supportive therapy, but as a potential cornerstone of precision medicine in neonatal seizure management. Rather than relying on a one-size-fits-all approach, treatment strategies should increasingly be guided by genetic profiling, metabolic screening, and biomarker-based diagnostics, all grounded in a growing understanding of neonatal neurochemistry. Vitamin B6-responsive epilepsies exemplify how individualized, mechanism-based treatments can dramatically improve both seizure control and neurodevelopmental outcomes. The path toward optimized neonatal seizure care lies in an integrated model that blends clinical acumen with emerging technologies—balancing rapid intervention with diagnostic precision. This approach not only enhances outcomes, but affirms our collective responsibility to protect the neurological futures of the most vulnerable: newborns at the very beginning of life.

## Figures and Tables

**Table 1:** Related diseases associated with vitamin B6 metabolism and precision therapy.

Disorder	Chromosomal Location of the Gene	Neurological Features	Treatment	Inheritance
PDE (OMIM #266100)	ALDH7A1, 5q23.2 (OMIM #107323)	Intractable neonatal seizures	Pyridoxine	AR
PNPO Deficiency (OMIM #610090)	PNPO, 17q21.32 (OMIM #603287)	Refractory neonatal seizures	PLP	AR
Hypophosphatasia (OMIM #241510)	ALPL, 1p36.12 (OMIM #171760)	Seizures, skeletal abnormalities	PLP, enzyme replacement	AR
Homocystinuria (OMIM #236200)	CBS, 21q22.3 (OMIM #613381)	Developmental delay, seizures	Pyridoxine, dietary management	AR

PDE: Pyridoxine-dependent epilepsy; ALDH7A1: aldehyde dehydrogenase 7 family member A1; PNPO: pyridox(am)ine 5’-phosphate oxidase; ALPL: alkaline phosphatase, liver/bone/kidney; CBS: cystathionine β-synthase; PLP: pyridoxal 5′-phosphate; AR, autosomal recessive.

**Table 2:** An overview of clinical features of vitamin B6-related neonatal epilepsies.

Feature	Description
Age of Onset	Typically, within the first days to weeks of life; atypical forms may appear later in infancy or even adolescence.
Seizure Types	Generalized tonic–clonic, focal clonic, myoclonic, tonic seizures, epileptic spasms; status epilepticus in ~8% of cases.
EEG Findings	Burst-suppression, hypsarrhythmia (especially in PNPO deficiency), interictal abnormalities; some cases show normal EEG.
Atypical Presentations	Dravet-like features, absence and atonic seizures, paroxysmal eye movements, response to folinic acid in some non-responders.
Neurodevelopmental Outcomes	Intellectual disability common, particularly expressive language delays; outcomes strongly linked to early diagnosis and treatment.
Prenatal Clues	Abnormal fetal movements reported in some cases, suggesting prenatal onset.

EEG, electroencephalographic; PNPO, pyridox(am)ine 5’-phosphatase oxidase.

**Table 3:** Treatment strategies for vitamin B6-responsive neonatal seizures.

Treatment	Indication/Notes
Pyridoxine (Vitamin B6)	First-line therapy for PDE; IV bolus (100 mg) in acute seizures; maintenance dosing varies by age.
PLP (Pyridoxal 5’-Phosphate)	Used in PNPO deficiency or pyridoxine non-responders; risk of hepatotoxicity.
Folinic Acid	Adjunctive in atypical cases unresponsive to pyridoxine alone.
Lysine-Restricted Diet	For ALDH7A1 deficiency (AQT deficiency); reduces toxic metabolite accumulation.
Arginine Supplementation	Reduces lysine transport into the brain; may improve motor and language outcomes.
Prenatal Pyridoxine	Recommended for at-risk pregnancies (100 mg/day); improves neurodevelopmental outcomes.

IV, intra-venous; PLP, pyridoxal 5’-Phosphate; PNPO, pyridox(am)ine 5’-phosphatase oxidase; ALDH7A1, aldehyde dehydrogenase 7 family member A1.

## Data Availability

No new data were created.
